# The Interplay between Tunneling and Parity Violation in Chiral Molecules

**DOI:** 10.3390/e26060456

**Published:** 2024-05-27

**Authors:** Daniel Martínez-Gil, Pedro Bargueño, Salvador Miret-Artés

**Affiliations:** 1 Fundación Humanismo y Ciencia, Guzmán el Bueno, 66, 28015 Madrid, Spain; daniel.martinez@ua.es; 2 Departamento de Física Aplicada, Campus de San Vicente del Raspeig, Universidad de Alicante, 03690 Alicante, Spain; pedro.bargueno@ua.es; 3 Instituto de Física Fundamental, Consejo Superior de Investigaciones Científicas, Serrano 123, 28006 Madrid, Spain

**Keywords:** tunneling, chiral molecules, parity violation

## Abstract

In this review, the concepts of quantum tunneling and parity violation are introduced in the context of chiral molecules. A particle moving in a double well potential provides a good model to study the behavior of chiral molecules, where the left well and right well represent the *L* and *R* enantiomers, respectively. If the model considers the quantum behavior of matter, the concept of quantum tunneling emerges, giving place to stereomutation dynamics between left- and right-handed chiral molecules. Parity-violating interactions, like the electroweak one, can be also considered, making possible the existence of an energy difference between the *L* and *R* enantiomers, the so-called parity-violating energy difference (PVED). Here we provide a brief account of some theoretical methods usually employed to calculate this PVED, also commenting on relevant experiments devoted to experimentally detect the aforementioned PVED in chiral molecules. Finally, we comment on some ways of solving the so-called Hund’s paradox, with emphasis on mean-field theory and decoherence.

## 1. Introduction

**Figure 1:**
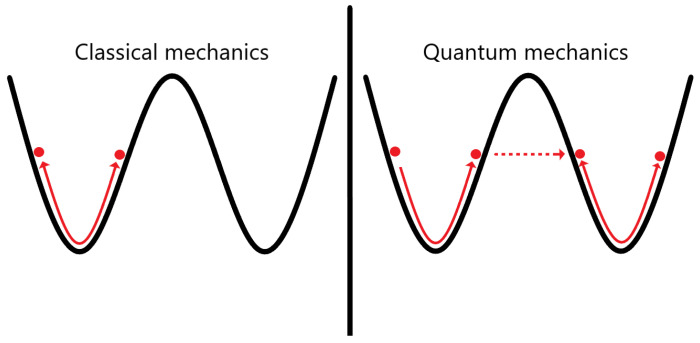
In this picture we can see the movement of an effective mass in an energy potential without friction in two cases: classical (**left**) and quantum (**right**). When the effective mass does not have enough energy to exceed the potential barrier, it oscillates around the minimum energy of the left well (classical case). In the the quantum case, the effective mass can go through the barrier by quantum tunneling. This representation serves as a toy model for studying tunneling effects in chiral molecules, where the left (**right**) well corresponds to the left (**right**) molecular enantiomer (see text for details).

Classical mechanics was developed in 1687 by Isaac Newton in his famous book *Philosophiae Naturalis Principia Mathematica* [1]. In the subsequent 200 years, it was used to theoretically interpret all known physical phenomena. In 1900, a set of discoveries related to the nature of light, molecules, and atoms led Max Planck to introduce a new way to describe the world: quantum mechanics (reviewed in [2]). In 1926, Erwin Schrödinger introduced the wave nature of matter [3], where a quantum wave function describes the state of a physical system following the famous Schrödinger equation

(1) $$i\hbar \frac{\partial }{\partial t}|\psi (t)\rangle =\hat{H}|\psi (t)\rangle ,$$

creating a probabilistic way to observe the world (the interested reader can consult a much more accurate discussion of the history of quantum mechanics in the excellent six-volume work of J. Mehra [4]). This probabilistic treatment gave place to one of the less intuitive and most important discoveries of all time: quantum tunneling (reviewed in [5]). Quantum tunneling is an effect where a particle can go through a potential barrier, in contrast with classical mechanics, where the particle must overcome this barrier by possessing enough energy to do it. This can be seen graphically in Figure 1.

In 1927, the German physicist Freidrich Hund [6,7], applied the Schrödinger Equation (1) to understand the behavior of chiral molecules, obtaining that tunneling has consequences on it, as we will see later. The term “chiral” was first introduced by Lord Kelvin in 1894 [8] to designate all geometric structures or groups of points whose reflections in a plane mirror cannot be superimposed with themselves. He called the two possible forms of those geometric structures “enantiomers”. Later, it was discovered that the fundamental symmetry that interconverts the enantiomers is parity symmetry (symmetry through a point of inversion), not reflection as Lord Kelvin stated [9]. Acquiring parity possesses great importance in the context of chiral molecules, especially parity-violating ones, as we will see in this manuscript.

Parity-violating processes were derived from the prediction by Lee and Yang [10], in which they postulated that parity can be violated in weak interactions, being the unique fundamental force capable of producing that behavior. Their prediction was confirmed one year later by Wu et al. [11].

The fact that the weak force violates parity made physicists to ask at what scales parity-violating effects could be present. The first answer came from Bouchiat and Bouchiat [12], by observing the spontaneous optical activity of bismuth atomic vapors, revealing effects of weak interactions in the atomic scale. After this important experiment was performed [13], Wood and coworkers discovered the nuclear anapole moment of cesium [14], with its different signal of parity violation, by improving low-temperature and high-resolution spectroscopic techniques. Therefore, parity violation was initially observed in elementary particles, and then in more complex systems like nuclei and atoms. If we continue considering more complex systems, we reach the molecular scale. Thus, one could ask if it makes sense to relate parity violation with molecular physics. This interesting interplay between chiral molecules, quantum tunneling and parity violation will be reviewed in this manuscript (the interested reader can find other reviews related to the topic of this work in refs. [15,16,17,18,19,20,21,22]).

Specifically, this review is organized as follows: In Section 2, we introduce a simple quantum treatment of chiral molecules and its relation with the tunneling time. In Section 3, we add parity violation to our description of chiral molecules, showing that there could exist an energy difference between the *L* and *R* enantiomers (PVED). In Section 4, we give some examples of parity-violating interactions, focusing on the electroweak force. In Section 5, we briefly review how the PVED can be calculated using simple or more sophisticated methods. In Section 6, we mention some experiments devoted to detecting the PVED. Possible solutions to the so-called “Hund’s paradox” are presented in Section 7. Finally, Section 8 provides the conclusions of the present work.

## 2. Introduction to Molecular Chirality

In the following section we will closely follow Quack’s works [23,24], trying to use a more modern formulation to clarify and emphasize the essentials.

The process of converting an *L* chiral molecule to its enantiomer, *R*, can be represented by the movement of an effective mass in an energy potential V(q), which is a function of a generalized coordinate, *q* [25]. If we use the time-independent Schrödinger, we can obtain the eigenfunctions, $\phi_{k}\left(q\right)$, and eigenvalues (energies), $E_{k}$, of the stationary states

(2) $$\begin{array}{cc} \hat{H}=\hat{T}+V\left(q\right)= & -\frac{\hbar^{2}}{2m}\nabla^{2}+V\left(q\right) \end{array}$$

(3) $$\begin{array}{cc} \hat{H}\phi_{k}\left(q\right)= & E_{k}\phi_{k}\left(q\right), \end{array}$$

where $\hat{T}$ is the kinetic energy, the potential energy is given by V(q), and $\hat{H}$ is the Hamiltonian operator.

In quantum mechanics, it is well known that the probability of the system to be in a determinate state can be expressed as

(4) $$P_{k}\left(q\right)={\left|\phi_{k}\left(q\right)\right|}^{2}.$$

Interestingly, Hund applied the time-dependent Schrödinger Equation (1) to the study of chiral molecules, with emphasis on the stereomutation process between left and right states driven by quantum tunneling. The time-dependent Schrödinger equation can be expressed as

(5) $$i\hbar \frac{\partial \psi (q,t)}{\partial t}=\hat{H}\psi \left(q,t\right),$$

and its general solution is

(6) $$\psi \left(q,t\right)=\sum_{k=1}^{\infty }c_{k}\phi_{k}\left(q\right)\exp\left\{-iE_{k}t/\hbar \right\},$$

where $c_{k}$ are complex coefficients.

As a first toy model, chiral molecules can be represented using only the two lowest energy states. Taking into account these two states, the solution of Equation (5) is

(7) $$\psi \left(q,t\right)=c_{+}|+\rangle \exp\left\{-iE_{+}t/\hbar \right\}+c_{-}|-\rangle \exp\left\{-iE_{-}t/\hbar \right\},$$

where we denote as |+〉 and |−〉 to the time-independent Hamiltonian eigenstates.

With this in mind, the probability density can be expressed as

(8) $$P\left(q,t\right)={\left|\psi (q,t)\right|}^{2}=\frac{1}{2}{||+\rangle +|-\rangle \exp\{-i\delta t/\hbar \}|}^{2},$$

where we choose that in the initial time the probability of finding both enantiomers is the same, so $c_{+}=c_{-}=\frac{1}{\sqrt{2}}$, and we define $\delta =E_{+}-E_{-}$. This result suggests that the system is located in the states |+〉 or |−〉 with a probability of

(9) $$\begin{array}{cccc} P_{-}\left(t\right)= & \sin^{2}\left(\delta t/\hbar \right), & P_{+}\left(t\right)= & \cos^{2}\left(\delta t/\hbar \right), \end{array}$$

therefore, the |+〉 and |−〉 states will oscillate with a period given by

(10) $$\tau =\frac{\hbar }{\delta }.$$

At this point, some comments are in order. It is well-known that chiral states are interconverted by the parity operator. The time-independent Hamiltonian eigenfunction |+〉 and |−〉 are also eigenfunctions of the parity operator ($\hat{P}|\pm \rangle =\pm |\pm \rangle$), but they have definite parity and, therefore, they cannot represent chiral molecules. As a consequence, we have to define a different algebraic base that can be used to represent chiral molecules.

**Figure 2:**
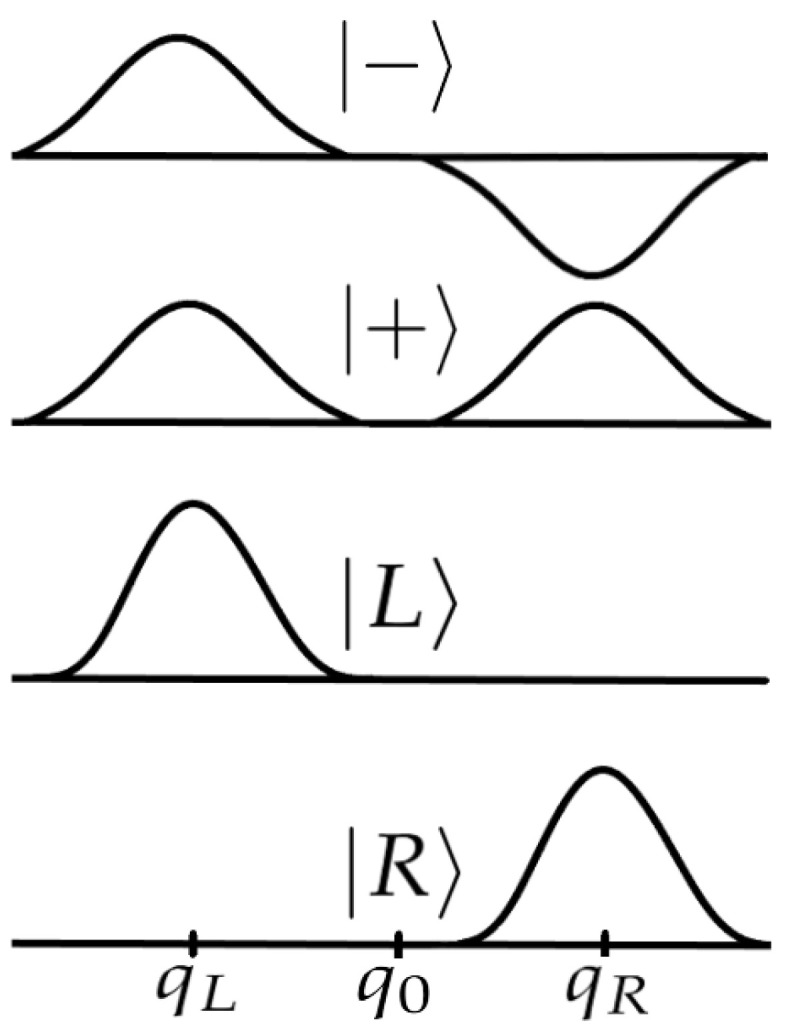
Representation of the states |+〉, |−〉, |L〉 and |R〉. Notice that the states |L〉 and |R〉 are only localized in $q_{L}$ and $q_{R}$, respectively, representing the *L* and *R* enantiomers (adapted from [23]).

The condition that has to be fulfilled is

(11) $$\begin{array}{cccc} \hat{P}|L\rangle = & |R\rangle , & \hat{P}|R\rangle = & |L\rangle , \end{array}$$

where we call |L〉 and |R〉 the states representing the *L* and *R* enantiomers. These states can be represented as

(12) $$\begin{array}{cc} |L\rangle = & \frac{1}{\sqrt{2}}\left(|+\rangle +|-\rangle \right) \end{array}$$

(13) $$\begin{array}{cc} |R\rangle = & \frac{1}{\sqrt{2}}\left(|+\rangle -|-\rangle \right), \end{array}$$

which are called chiral states or chiral base (see Figure 2)). These states are not eigenstates of the Hamiltonian, being delocalized and having no defined energy (so it is necessary to work using the average of the energy). With this in mind, it is easy to reach an expression similar to Equation (10), and conclude that chiral molecules undergo an oscillating motion between *L* and *R* enantiomers, with a period of

(14) $$\tau_{LR}=\frac{\hbar }{2\delta }.$$

With this in mind, we would like to remark that Hund performed two important considerations. First, he realized that although the energies $E_{+}$ and $E_{-}$ were not enough to exceed the potential barrier V(q), the transition between enantiomers could be observed, which is impossible in classical mechanics. This fact was the origin of the recognized tunneling effect in molecular physics, as we mentioned in the introduction. The second consideration is related to what is known as “Hund’s paradox” [7], as pointed out by Harris and Stodolsky in [26]. This paradox refers to the stability of some molecules in one specific enantiomeric state (e.g., CHFClBr, amino acids, and sugars), which is in principle impossible because there must be a periodic transition between enantiomers, in agreement with Equation (14). This paradox can be solved, as first noted by Harris and Stodolsky in 1978 [26] by the introduction of a new concept into the model: parity violation (let us remark that the relation of parity violation to Hund’s interpretation was also described and discussed in some detail in [24]). However, there are different ways of resolving the paradox, as we will see in Section 7.

## 3. Parity Violation

**Figure 3:**
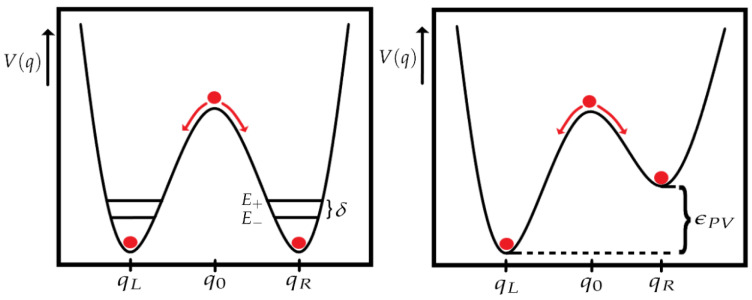
In both pictures, we can observe a double well potential. In the left picture, the role of the δ parameter is represented. Even in the absence of parity violation, the states |+〉 and |−〉 have different energies giving place to the tunneling time. In the right picture, the effect of adding parity violation to our system is shown. The potential well can be asymmetrical, giving place to different energies between the left and right wells, defined with the parameter $\epsilon_{PV}$ (adapted from [23,24]).

We have described chiral molecules in a two-state system without parity violation, described as a symmetric double well. We can now add parity violation to our model, which can be represented by an asymmetric double well, modifying the Hamiltonian [26], which is expressed as

(15) $$\hat{H}={\hat{H}}^{0}+{\hat{H}}^{PV}=\left(\begin{array}{cc} E_{0} & \delta \\ \delta & E_{0} \end{array}\right)+\left(\begin{array}{cc} -\epsilon_{PV} & 0\\ 0 & \epsilon_{PV} \end{array}\right),$$

where ${\hat{H}}_{PV}$ is the parity-violating term of the Hamiltonian ($\hat{P}H^{PV}{\hat{P}}^{-1}=-H^{PV}$), allowing the breakdown of the enantiomer degeneration. Additionally, $E_{0}$, δ, and $\epsilon_{PV}$ are defined as

(16) $$\begin{array}{cccccc} E_{0}= & \frac{H_{LL}+H_{RR}}{2}, & \delta = & H_{RL}, & \epsilon_{PV} & =\frac{H_{LL}-H_{RR}}{2}, \end{array}$$

where we used the notation $H_{XY}=\langle X|H|Y\rangle$. The δ parameter is defined in the previous section, and it is related to the height of the potential well and the tunneling time ($\delta =E_{+}-E_{-}$), and $\epsilon_{PV}$ is related to the energy difference between the two potential wells. The $\epsilon_{PV}$ parameter is also known as PVED (Parity Violating Energy Difference) and represents the energy difference between the *L* and the *R* enantiomers (see Figure 3).

We will define |A〉,|B〉 as the eigenstates of the Hamiltonian (15). It is remarkable that |A〉,|B〉 are different from the definite parity states |+〉 and |−〉, and they can be expressed in the function of the chiral states as [27,28]

(17) $$\left(\begin{array}{c} |A\rangle \\ |B\rangle \end{array}\right)=\left(\begin{array}{cc} \sin\theta & \cos\theta \\ \cos\theta & -\sin\theta \end{array}\right)\left(\begin{array}{c} |L\rangle \\ |R\rangle \end{array}\right),$$

where θ is the mixing angle and can be obtained as

(18) $$\tan2\theta =\frac{2H_{RL}}{H_{LL}-H_{RR}}=\frac{\delta }{\epsilon_{PV}}.$$

The energies of the system are now given by the eigenstates of the Hamiltonian (17) and they are

(19) $$E_{AB}=E_{0}\mp \Delta ,$$

with

(20) $$\begin{array}{c} \Delta \equiv \sqrt{\epsilon_{PV}^{2}+\delta^{2}}. \end{array}$$

It is interesting to calculate the probability of a chiral molecule being located in its left or right state. For that, we have to express the wave function in the chiral base, obtaining

(21) $$|\psi (t)\rangle =c_{L}\left(t\right)|L\rangle +c_{R}\left(t\right)|R\rangle .$$

The evolution of $c_{L}\left(t\right)$ and $c_{R}\left(t\right)$ will be determined by the time-dependent Schrödinger Equation (1):

(22) $$i\hbar \partial_{t}\left(\begin{array}{c} c_{L}\left(t\right)\\ c_{R}\left(t\right) \end{array}\right)=\left(\begin{array}{cc} E_{0}-\epsilon_{PV} & \delta \\ \delta & E_{0}+\epsilon_{PV} \end{array}\right)\left(\begin{array}{c} c_{L}\left(t\right)\\ c_{R}\left(t\right) \end{array}\right).$$

If we suppose that $E_{0}=0$, $c_{L}\left(0\right)=1$ and $c_{R}\left(0\right)=0$, we obtain

(23) $$\begin{array}{cc} c_{L}\left(t\right)= & \cos\left(\frac{\Delta }{\hbar }t\right)-\frac{i\epsilon_{PV}}{\Delta }\sin\left(\frac{\Delta }{\hbar }t\right), \end{array}$$

(24) $$\begin{array}{cc} c_{R}\left(t\right)= & -\frac{i\delta }{\Delta }\sin\left(\frac{\Delta }{\hbar }t\right), \end{array}$$

with $|c_{L}\left(t\right){|}^{2}$ and $|c_{R}\left(t\right){|}^{2}$ being the probability of the wave function to be in the |L〉 or |R〉 states, respectively. At this point, it is necessary to remark two specific cases [27,28]:

**Case 1,** θ→0:

This case implies that $\epsilon_{PV}\gg \delta$ (tan2θ→0) tending the energy eigenstates to be the chiral states

(25) $$\begin{array}{cccc} |A\rangle \to & |L\rangle , & |B\rangle \to & |R\rangle . \end{array}$$

As we know, the states |A〉 and |B〉 are eigenstates of the Hamiltonian (17), therefore they are localized states. In this particular case, the |L〉 and |R〉 states are also localized, with the chiral molecule being stable in one of the two possible enantiomers. According to (23), $|c_{L}\left(t\right){|}^{2}=1$, with the chiral molecule being stable in the *L* state, solving the aforementioned Hund’s paradox, as pointed out by Harris and Stodolsky [26].

**Case 2** θ→π/4:

This case implies that $\epsilon_{PV}\ll \delta$ (tan2θ→∞), making sense to recover the non-parity violating case. If we use the Equation (17), and we impose θ=π/4, we obtain the Equations (12) and (13), resulting in

(26) $$\begin{array}{cccc} |A\rangle & =|+\rangle , & |B\rangle & =|-\rangle . \end{array}$$

**Figure 4:**
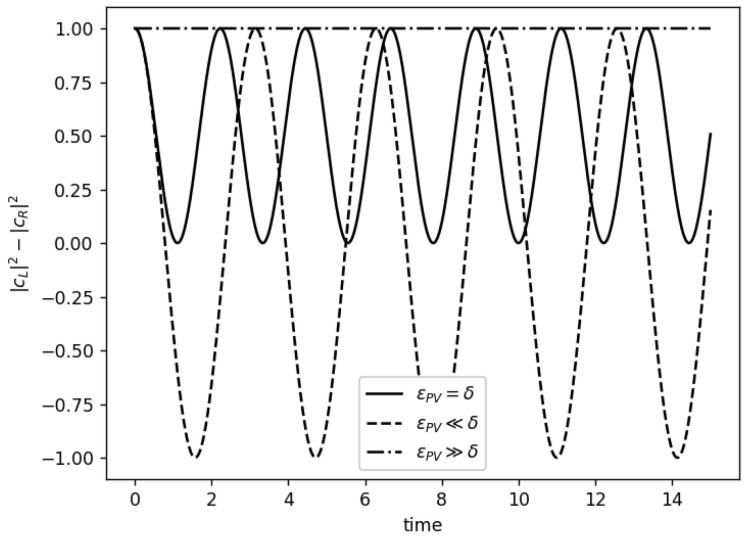
In this figure, the difference between the probability of the *L* and *R* states is represented as a function of the time. As we increase the value of $\epsilon_{PV}$ (or decrease the value of δ), the wave function tends to be localized in the *L* state, thus providing a possible solution to Hund’s paradox (we plot $\epsilon_{PV}=100\;\delta$ to show this behavior). On the contrary, if $\epsilon_{PV}\ll \delta$ ($\delta =100\;\epsilon_{PV}$ in our case), the symmetric oscillatory behavior of the non-parity violation case is recovered.

As the eigenstates of the Hamiltonian $\hat{H}$ (15) are equal to the eigenstates of ${\hat{H}}_{0}$, we can conclude that there is no parity violation in this case. Both cases can be better understood with the help of Figure 4.

At this point, some comments are in order. As we have seen, if parity violation affects our system, there could be an energy difference between enantiomers $\epsilon_{PV}$. In the other case, enantiomers are degenerate ($\epsilon_{PV}=0$). This behavior can be seen algebraically as follows:

**Without parity violation**

We already know the expressions for |L〉 and |R〉 without parity violation:

(27) $$\begin{array}{cc} |L\rangle = & \frac{1}{\sqrt{2}}\left(|+\rangle +|-\rangle \right) \end{array}$$

(28) $$\begin{array}{cc} |R\rangle = & \frac{1}{\sqrt{2}}\left(|+\rangle -|-\rangle \right). \end{array}$$

With this in mind, we can perform the next development

$$\begin{array}{cc} E_{L}=\langle L|\hat{H}|L\rangle = & \frac{1}{2}\left(\langle +|+\langle -|\right)\hat{H}\left(|+\rangle +|-\rangle \right)=\frac{1}{2}\left(\langle +|\hat{H}|+\rangle +\langle -|\hat{H}|-\rangle \right)=\frac{1}{2}\left(E_{+}+E_{-}\right)\\ E_{R}=\langle R|\hat{H}|R\rangle = & \frac{1}{2}\left(\langle +|-\langle -|\right)\hat{H}\left(|+\rangle -|-\rangle \right)=\frac{1}{2}\left(\langle +|\hat{H}|+\rangle +\langle -|\hat{H}|-\rangle \right)=\frac{1}{2}\left(E_{+}+E_{-}\right), \end{array}$$

where we defined $\langle +|\hat{H}|+\rangle =E_{+},\langle -|\hat{H}|-\rangle =E_{-}$. Therefore we have shown that $E_{L}=E_{R}$ ($\epsilon_{PV}=0$), confirming the enantiomer degeneration in the absence of parity violation.

**With parity violation**

In a parity-violating context, the chiral states can be represented in function of the Hamiltonian eigenstates as

(29) $$\begin{array}{cc} |L\rangle = & \sin\theta |A\rangle +\cos\theta |B\rangle \end{array}$$

(30) $$\begin{array}{cc} |R\rangle = & \cos\theta |A\rangle -\sin\theta |B\rangle . \end{array}$$

A process similar to the previous one can be performed:

$$\begin{array}{cc} E_{L}=\langle L|\hat{H}|L\rangle = & \left(\cos\theta \langle A|+\sin\theta \langle B|\right)\hat{H}\left(\cos\theta |A\rangle +\sin\theta |B\rangle \right)=E_{A}\cos^{2}\theta +E_{B}\sin^{2}\theta \\ E_{R}=\langle R|\hat{H}|R\rangle = & \left(\sin\theta \langle A|-\cos\theta \langle B|\right)\hat{H}\left(\sin\theta |A\rangle -\cos\theta |B\rangle \right)=E_{A}\sin^{2}\theta +E_{B}\cos^{2}\theta . \end{array}$$

If we use Equation (19), we arrive to

(31) $$\begin{array}{cc} E_{L}= & E_{0}+\Delta \left(\sin^{2}\theta -\cos^{2}\theta \right), \end{array}$$

(32) $$\begin{array}{cc} E_{R}= & E_{0}-\Delta \left(\sin^{2}\theta -\cos^{2}\theta \right). \end{array}$$

Therefore, the energy of the enantiomers is not the same ($\epsilon_{PV}=\Delta \left(\sin^{2}\theta -\cos^{2}\theta \right)$) unless for $\theta =\frac{\pi }{4}\pm n\frac{\pi }{2}$∀*n*∈N where $\epsilon_{PV}=0$.

In this section, we have mainly reviewed Harris and Stodolsky [26] and Quack’s works [24] in order to show in some detail, using a modern Dirac bra/ket formalism how parity violation affects the energetics of chiral molecules, resulting sometimes in their apparent stability. Although tunneling is well-known theoretically and experimentally, there is still interest in applying novel theories in this framework, like instanton calculations, which predicts quantum tunneling ab initio [29]. On the contrary, the PVED has not yet been detected, its relationship with molecular chirality constituting one of the most intriguing problems of our time [30]. To give the reader an idea of the importance of finding the PVED, in [30] it is compared with finding the Higgs boson (together with spotting distant signs of life, looking for extra dimensions, catching a gravitational wave, and redefining the kilogram). Before going into a brief review of theory and experiments designed to study parity violation in chiral molecules, we will give a bird’s eye view of the type of interactions that can show a parity-violating behavior.

## 4. Parity-Violating Interactions

As was said before, weak interaction is a unique fundamental force capable of producing parity violation in the Standard Model of Particle Physics (SMPP). Despite that, there are other relevant theories beyond the SMPP that can violate parity, like axion interactions or modified gravity theories, which we would like to mention briefly.

The axion is a particle not included in the SMPP, being a dark matter candidate (see [31] for a recent review). A parity-violating electron–nucleon interaction mediated by an axion was proposed by Moody and Wilczek [32] and is given by

(33) $$H_{ax}=\left(g_{s}^{N}g_{p}^{e}\right)\frac{{\vec{\sigma }}_{e}\cdot \vec{r}}{8\pi m_{e}}\left(\frac{m_{\phi }}{r}+\frac{1}{r^{2}}\right)e^{-m_{\phi }r},$$

where c=ℏ=1, $g_{s}^{N}$ is the scalar coupling constant of the axion to a nucleon, $g_{p}^{e}$ is the pseudoscalar coupling constant to the electron, $\vec{r}$ represents the separation vector between the electron and the nucleon, and $m_{\phi }$ is the mass of the axion. Interestingly, P-odd interactions of electrons with scalar cosmic fields (like axions) behave similarly to interactions due to an electroweak coupling of electrons and nucleons in chiral molecules, as shown in ref. [33].

Other different parity-violating mechanisms can be included in gravity. As commented in [34], Leitner and Okubo were the first who pioneered this idea [35]. After their proposal, Hari Dass wrote a parity-violating gravitational potential of the form (c = 1) [36]

(34) $$V^{Grav}\left(r\right)=GM\left(\alpha_{1}\frac{\vec{s}\cdot \vec{r}}{r^{3}}+\alpha_{2}\frac{\vec{s}\cdot \vec{v}}{r^{2}}+\alpha_{3}\frac{\vec{s}\times \vec{r}\cdot \vec{v}}{r^{3}}\right),$$

where *M* represents the mass of the gravitating object, $\vec{r}$ is the separation vector from that mass to the test particle, whose spin and velocity are given by $\vec{s}$ and $\vec{v}$, respectively.

Another relevant proposal is the Chern–Simons (CS) theory for gravity [37], extending general relativity by considering not only the Einstein tensor but also the C-tensor [38] and an extra pseudoscalar field (a field which violates parity).

We think it is important to remark that it is not possible to give a precise value of the PVED due to an interaction beyond the SMPP because, in general, the relevant coupling constants are unknown parameters. However, by employing non-conlusive searches of electroweak PVED in chiral molecules it is possible to bound these unknown coupling constants (as was already done in [39,40] for modified gravity theories and in [33,41] for cosmic fields).

Although there are other relevant theories, in the following lines we will only consider electroweak interactions, which are routinely included in quantum chemistry calculations to study the PVED in chiral molecules.

### Electroweak Interaction

At the beginning of the discovery of the weak force, it was believed that it depended on charge transference (charged currents). Subsequently, in some attempts to unify weak and electromagnetic forces [42,43,44], a new type of interaction emerged, based on weak neutral currents, which were capable of producing observable effects of parity violation in atoms [12,45]. With this in mind and knowing that molecules are essentially made by nucleons and electrons (only stable nuclei are considered), we can use Quantum Field Theory and Feynman’s rules to propose a Hamiltonian that produces PVED in chiral molecules. This Hamiltonian can be derived as follows:

It is well-known that the weak neutral current Hamiltonian can be expressed as

(35) $$H_{NC}=-:\frac{G_{F}}{\sqrt{2}}J_{\mu }J^{\mu }:,$$

where $J_{\mu }$ are the weak currents and $G_{F}$ is the Fermi constant and the notation :X: refers to the normal ordering of the *X* operators. The weak currents can be formed by a hadronic and a leptonic part

(36) $$J_{\mu }=J_{\mu }^{h}+J_{\mu }^{l}.$$

**Figure 5:**
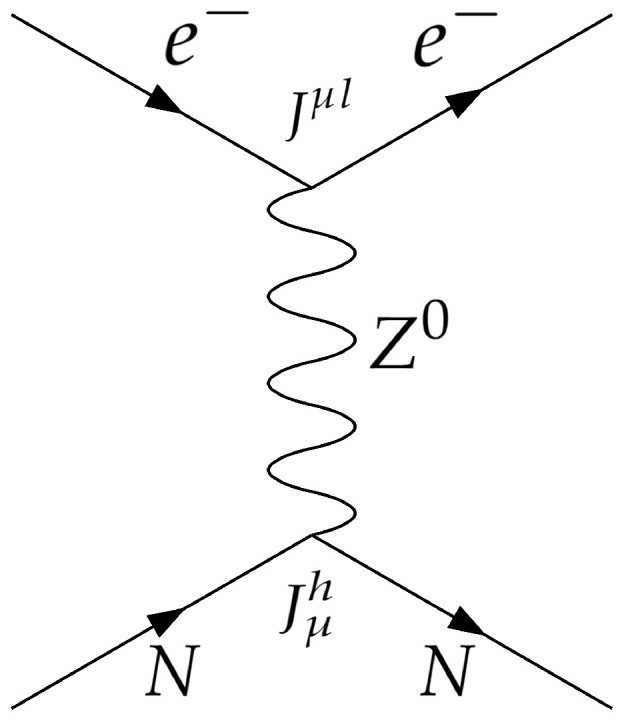
Feynman diagram of an electron-nucleon interaction mediated by a $Z^{0}$ boson.

Specifically, we are interested in an electron–nucleon (lepton–hadron) interaction, given by the Feynman diagram shown in Figure 5, arriving to

(37) $$H_{NC}=-:\frac{G_{F}}{\sqrt{2}}J_{\mu }^{h}J^{\mu l}:.$$

Applying Feynman’s rules to obtain the weak currents, we arrive to the electron–nucleon interaction Hamiltonian mediated by a $Z^{0}$ boson (the propagator of the weak neutral current), which can be expressed as

This Hamiltonian represents the interaction between electrons and nucleons of the chiral molecule, mediated by a $Z^{0}$ boson (the propagator of the weak neutral current), and can be expressed as

(38) $$H_{NC}=\frac{G_{F}}{\sqrt{2}}:\bar{N}\left(x\right)\gamma^{\mu }\left(V^{N}-A^{N}\gamma^{5}\right)N\left(x\right)\bar{e}\left(x\right)\gamma_{\mu }\left(V^{e}-A^{e}\gamma_{5}\right)e\left(x\right):,$$

where $G_{F}$ is the Fermi constant, $\gamma^{\mu }$ are the Dirac matrices which satisfy $\left\{\gamma^{\mu },\gamma^{\nu }\right\}=2\eta^{\mu \nu }$ ($\eta^{\mu \nu }$ is the Minkowskian metric with signature (−,+,+,+)), $\bar{\psi }\equiv \psi^{†}\gamma^{0}$ is Dirac adjoint, $A^{e}$, $A^{N}$, $V^{e}$, and $V^{N}$ are coupling constants, e(x) and N(x) are the electron and nucleon fields respectively.

Although non-relativistic calculations are not working well for heavy-element molecules, it is interesting to consider the non-relativistic limit [12,16,46] to gain some intuition on what is going on. Note that, in order to obtain the aforementioned non-relativistic Hamiltonian, some approximations must be made. In this regime, the Dirac matrices can be expressed as

(39) $$\gamma^{0}=\left(\begin{array}{cc} I_{2} & 0\\ 0 & -I_{2} \end{array}\right),\;\;\;\;\;\gamma^{k}=\left(\begin{array}{cc} 0 & \sigma_{k}\\ -\sigma_{k} & 0 \end{array}\right),\;\;\;\;\;\gamma^{5}=\left(\begin{array}{cc} 0 & -I_{2}\\ -I_{2} & 0 \end{array}\right),$$

where $\sigma_{k}$ are the Pauli matrices and $I_{2}$ is the 2×2 identity matrix, and the spatial part of a biespinor is

(40) $$u\left(\vec{r}\right)=\left(\begin{array}{c} \chi_{s}\left(\vec{r}\right)\\ \frac{\vec{\sigma }\cdot \vec{p}}{2m}\chi_{s}\left(\vec{r}\right) \end{array}\right),$$

where $\vec{r}$ is the position vector in spherical coordinates and $\chi_{s}$ are the two possible fermion spinors

(41) $$\chi_{1}=\left(\begin{array}{c} 1\\ 0 \end{array}\right),\;\;\;\;\;\chi_{2}=\left(\begin{array}{c} 0\\ 1 \end{array}\right).$$

It also has to be assumed that the mass of the nucleons is high compared with that of the electrons, arriving to

(42) $$N\left(\vec{r}\right)\approx \left(\begin{array}{c} \chi_{s}\left(\vec{r}\right)\\ 0 \end{array}\right).$$

If the number of protons and nucleons are of the same order we arrive, after considering Equation (42), to

$$\bar{N}\left(\vec{r}\right)\gamma^{0}N\left(\vec{r}\right)=\langle \delta (\vec{r})\rangle \approx \left(Zg_{V}^{p}+Ng_{V}^{N}\right)$$

(43) $$=\frac{1}{2}\left[(1-4\sin^{2}\theta_{W})Z-N\right]\delta \left(\vec{r}\right)=\frac{1}{2}Q_{W}\delta \left(\vec{r}\right),$$

where the value of the coupling constants are

(44) $$g_{V}^{p}=-g_{V}^{n}=\frac{1}{2}\left(1-4\sin^{2}\theta_{w}\right).$$

The weak charge, $Q_{W}$, is given by

(45) $$Q_{W}=\left(1-4\sin^{2}\theta_{W}\right)Z-N,$$

where $\theta_{W}$ is the Weinberg angle and *N* and *Z* are the number of neutrons and protons, respectively. With all these approximations in mind, it is easy to arrive at the non-relativistic electron–nucleon interaction Hamiltonian [12,16,46]

(46) $$H_{NC}=\frac{G_{F}}{4\sqrt{2}}\sum_{j}\sum_{i}Q_{W_{j}}\left\{{\vec{p}}_{i}\cdot {\vec{\sigma }}_{i},\delta \left(\vec{r_{j}}-{\vec{r}}_{i}\right)\right\},$$

where $\vec{p}$ and $\vec{\sigma }$ are the momentum and spin of the electrons, ρ is the nucleon density and the index *i* and *j* refers to a summation over electrons and nucleons, respectively.

In order to understand why this Hamiltonian is capable of producing PVED in chiral molecules, two concepts have to be introduced: *true chirality* and *false chirality* [47,48,49]. Barron stated that “true chirality is possessed by systems that exist in two distinct enantiomeric states that are interconverted by space inversion but not by time reversal combined with any proper spatial rotation” [48]. On the contrary, false chirality occurs in systems that exist in two different enantiomeric states, which are interconverted by spatial inversion as well as time inversion followed by spatial rotation. In terms of fundamental symmetries, a Hamiltonian is truly chiral when it respects both parity and time inversion symmetries and is falsely chiral when it respects parity and violates time inversion. We remark that these two definitions are of great importance in the context of this review because only a truly chiral Hamiltonian can produce PVED in chiral molecules [48] (this have been recently demonstrated in ref. [50] within a QFT formalism). Following Barron’s definitions, the $\vec{p}\cdot \vec{\sigma }$ term appearing in the electroweak Hamiltonian of Equation (46) is truly chiral, and therefore it could produce PVED in chiral molecules.

## 5. Calculations of PVED

Although the Hamiltonian (46) violates parity, there is still a problem in calculating the PVED. In the non-relativistic approximation, the molecular wave function is always real, while the parity-violating part of the Hamiltonian (46) is purely imaginary. This led to a zero expectation value for $H_{NC}$ over the molecular wave function. There are different possible solutions to this problem. Here, for brevity, we will only mention three of them: (i) adding a parity-odd perturbation operator into a fully relativistic four-component Dirac–Hartree–Fock framework treatment of the parity-violating energy differences in chiral molecules [51]; (ii) using a multiconfiguration linear response approach to electroweak quantum chemistry including effects from the parity-violating weak nuclear force [52]; and (iii) maintaining a non-relativistic regime, adding a spin-orbit coupling to give a first-order correction to the wave function [53] (in order to highlight the need for spin-orbit coupling, note that the non-relativistic wave function is also an eigenstate of $S_{z}$ and that the parity-violating operator is a triplet operator (we acknowledge one of the anonymous referees for this comment.)). This spin-orbit coupling (which only works well for elements up to at most the 5th period) can be written as

(47) $$V^{SO}=\frac{1}{4}\alpha^{2}\sum_{i}\left(E_{i}\times p_{i}\right)\cdot \sigma_{i},$$

where α is the fine-structure constant and $E_{i}$ is the electric field seen by an electron *i*. Then, the ground state $\psi_{0}$ is perturbed as

(48) $$\psi_{0}\to \psi_{0}+\sum_{n}\frac{\langle n|V^{SO}|n\rangle }{E_{0}-E_{n}}\psi_{n},$$

resulting in a term for the PVED different from 0, which is

(49) $$\Delta E_{PV}=\sum_{n}\frac{\langle 0|H^{EW}|n\rangle \langle n|V^{SO}|0\rangle }{E_{0}-E_{n}}+C.C.$$

At this point, an estimation of the PVED can be done. In one way, this estimation was performed in a work by Bouchiat and Bouchiat [12]. In that work, they stated that, for dominant heavy atoms with atomic number *Z*, the $H^{EW}$ part of the Equation (49) is of order $GZ^{3}\alpha$. By the other way, from atomic theory, we know that the $V^{SO}$ part of Equation (49) is of order $Z^{2}\alpha^{2}$. With these considerations in mind, we can estimate that

(50) $$\Delta E_{PV}\approx G\alpha^{3}Z^{5},$$

which shows that the higher *Z*, the higher the PVED. The Equation (50) is usually known as $Z^{5}$ scaling law, and it was used in numerical calculous in different works [54,55].

Although nowadays there are other methodologies based on different levels for the theory employed, we would like to remark the CIS-RHF formalism [56,57], a formalism that emerged from critically analyzing calculations of electroweak quantum chemistry. This formalism is a perturbation-theory mixture of the ground RHF (restricted Hartree-Fock) with the CIS (configuration interaction singles) excited states. Using this approach, the authors found that the PVED in chiral molecules has essentially a tensor character

(51) $$\epsilon_{PV}=\mathrm{Tr}\{\epsilon_{PV}^{i,j}\}=\epsilon_{PV}^{xx}+\epsilon_{PV}^{yy}+\epsilon_{PV}^{zz},$$

which transforms as a polar vector in its first index and as an axial vector in its second index, this tensor being a pseudoscalar one. With this method in mind, the authors of refs. [52,56,57] calculated $\epsilon_{PV}$ in typical chiral molecules, obtaining a PVED up to two orders of magnitude higher than using the widely used SDE-RHF method [53]. Please note that these enhancements have been confirmed by other groups in subsequent years [51,58,59,60].

Finally, we would like to remark that there are other interesting methods to mention. For example, if we want to study parity-violating potentials in chiral molecules with light nuclei, an interesting way is using the Breit–Pauli approximation [61]. On the contrary, if molecules involving heavy atoms are considered, the Dirac–Fock theory is appropriate [51,60,62]. Alternatively, other efficient two-component (quasi-relativistic) methods are available, such as the zeroth-order regular approximation approach (ZORA) [63,64], the Douglas–Kroll–Hess method (DKH) [65] or the X2C method [66], which could help to study effects higher than zero order. For the interested reader, different methods to calculate the PVED together with an exhaustive list of references can be found in [21].

## 6. Experiments Searching the PVED

It is remarkable that although the search for PVEDs has been exhaustive work, it has not yet been detected, due to the high sensitivity needed in the experiments. Up to this date, in our opinion, there are two ongoing experiments that are more than 25 years in the making whose purpose is the detection of PVED in chiral molecules. One of them is located in Zürich, based on a proposal made by Quack in 1986 [23] and the other one emerged in Paris in 1999 [67] (see [25] if more information is required). As an incomplete list, we would like to remark on other experimental approaches working on parity-violating effects on chiral molecules [68,69,70,71,72,73,74,75,76,77,78] (see [15,79] for more information about other experiments). Other interesting techniques have been recently proposed in the last few years [80,81,82,83,84,85]. Here, we will briefly comment on the Zürich and Paris proposals.

Firstly we will discuss the Zürich experiment, mainly developed before 1986 by Quack’s group [23]. They use excited energy levels of achiral molecules with well-defined parity that connect both enantiomers by radiative electric dipole transition moments. They prepare coherent superpositions of parity-defined states in the fundamental state using those energy levels, following the temporal evolution of the molecular parity. From this, $\epsilon_{PV}$ can be obtained directly, which is the objective of the experiment (see Section 4.2 of the review [25] for more detailed information). Recently, they have achieved an experimental sensitivity (using $NH^{3}$) of $\epsilon_{PV}=100\;aeV$ [86]. Such values have been found theoretically in chiral molecules with lighter nuclei than sulfur and chlorine [19,87,88], so maybe such data can be of great interest to future experiments. At this point we would like to note that, although Harris proposed [89] that electric field optical activity could provide a different way of measuring dynamics created by parity violations for short times, the use of real superpositions of chiral amplitudes [90,91,92] seems mandatory. As Harris stated in [89], the idea of measuring electric field optical activity could be in principle implemented in Quack’s proposal [23].

The other experiment was proposed by Letokhov in 1975 and 1976 [93,94]. This experiment tries to measure the differences in the high-resolution spectrum in separate *L* and *R* enantiomers, which can have a transition frequency $\nu_{L}$, $\nu_{R}$ for specific transitions. The energy difference between two molecular levels can be expressed in function of the transition frequency as

(52) $$\epsilon_{PV}^{*}-\epsilon_{PV}=h\left(\nu_{R}-\nu_{L}\right).$$

The group of the Laboratoire de Physique des Lasers in Paris achieved a sensitivity of $\frac{\Delta \nu^{PV}}{\nu }\approx 10^{-13}$ [67]. Furthermore, it is expected that, for specific molecules composed of Ruthenium (Ru) and Osmium (Os) atoms, measurement could be in the order of $\frac{\Delta \nu^{PV}}{\nu }\;\approx \;10^{-14}$ [95]. Therefore, we think we are close to observing the PVED in chiral molecules.

We would like to close this section by briefly commenting other newer developments in the study of parity violation in chiral molecules. We would like to mention [96], where it has been shown that the search for P-odd signals in molecules can benefit from the long interrogation times accessible in trapped chiral molecular ions. In particular, the calculations performed in [96] have revealed that ${\mathrm{CHDBrI}}^{+}$ and ${\mathrm{CHCaBrI}}^{+}$, among others, are favorable candidates to study PVED in chiral molecules (in [97]; the authors also demonstrate the promising potential of CHDBrI^+^). Regarding rotational spectroscopy, the authors of [85] have been able to predict the relative shifts in rotational constants that are induced by electroweak parity violation in the equilibrium structure of a chiral transition metal complex, showing that relative differences between rotational constants are of the order of $10^{-14}$, which is a favorably large effect. A framework to perform PV precision measurement in a racemic sample of chiral molecules has been introduced in [84]. Interestingly, the authors show that it is readily applicable to existing experiments using vibrational spectroscopy. Finally, as a new promising option to measure PVED in chiral molecules, the authors of [98] proposed to use tailored microwave fields as a way to control quantum internal states of chiral molecules.

## 7. Solutions to Hund’s Paradox

**Figure 6:**
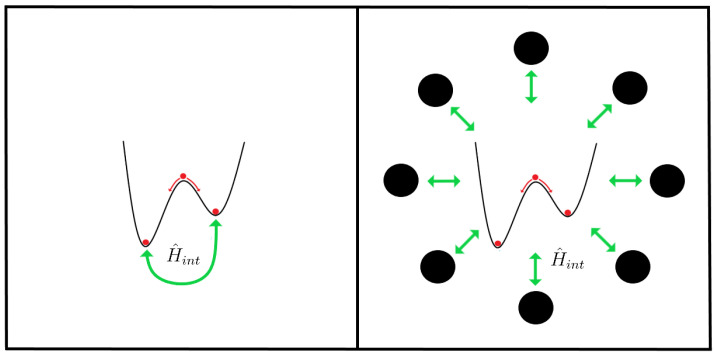
This figure is divided into two parts, each one corresponding to one form of solving Hund’s paradox. In the left part, in green color, the non-linear intramolecular interaction between the L and R enantiomer is represented. In the right part, the green color represents the interaction between the chiral molecule and the environment. See text for details.

As a last part of this review, we will continue the discussion about solutions to Hund’s paradox from Section 2, specifically focusing on two possibilities. The first one uses what is known as mean-field theory [99,100,101], adding a non-linear interaction term between the L and R enantiomer to the Hamiltonian. The other one employs decoherence theory [26,102,103,104,105,106,107,108,109,110,111,112], which takes into account the interaction between the chiral molecule and the environment. These two ways of solving Hund’s paradox can be better understood with Figure 6.

### 7.1. Mean-Field Theory

Following Vardi [99] and Grecchi and Sacchetti [101], and keeping the formalism of the two-level system Hamiltonian given by Equation (15), non-linear intramolecular interactions, represented by ${\hat{H}}_{int}$, can be introduced using the following Hamiltonian

(53) $$\hat{H}={\hat{H}}_{0}+{\hat{H}}_{PV}+{\hat{H}}_{int}=\left(\begin{array}{cc} E_{0} & \delta \\ \delta & E_{0} \end{array}\right)+\left(\begin{array}{cc} -\epsilon_{PV} & 0\\ 0 & \epsilon_{PV} \end{array}\right)+\left(\begin{array}{cc} F(c_{L},c_{R}) & G(c_{L},c_{R})\\ -G(c_{L},c_{R}) & -F(c_{L},c_{R}) \end{array}\right),$$

where $F(c_{L},c_{R})$ and $G(c_{L},c_{R})$ are specific functions which depends on $c_{L}\left(t\right)$ and $c_{R}\left(t\right)$.

As pointed out by Vardi [99] and Bargueño et al. [110], a specific intramolecular interaction Hamiltonian can be expressed as

(54) $$\hat{H}=\left(\begin{array}{cc} \epsilon_{PV}+\frac{\kappa }{2}\left(\right|c_{L}{|}^{2}-|c_{R}{|}^{2}) & \delta \\ \delta & -\epsilon_{PV}-\frac{\kappa }{2}\left(\right|c_{L}{|}^{2}-|c_{R}{|}^{2}) \end{array}\right),$$

where κ represents the strength of the non-linear intramolecular interactions.

**Figure 7:**
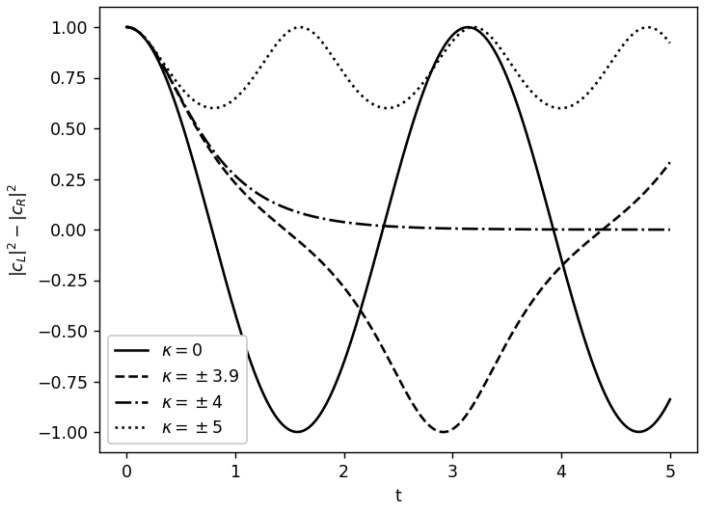
In this figure, the difference between the probability of the L and R states with non-linear intramolecular interactions, κ, is represented as a function of the time, for different values of κ. We take $\epsilon_{PV}=0$, $c_{L}\left(t=0\right)=1$, $c_{R}\left(t=0\right)=0$. This figure is based on Figure 2 of Vardi’s work [99].

Let us now briefly comment on the physical role of this new κ parameter. In order to do that, we solve the time-dependent Schrödinger Equation (5) using (54). Figure 7 shows the difference between the probability of the L and R states. In this case, we do not take into account the PVED ($\epsilon_{PV}=0$) to remark that this model can solve Hund’s paradox without parity violation. As Vardi exposed [99], in the linear limit (represented by the solid line) harmonic tunneling oscillations between the L and R enantiomers are observed. These oscillations become anharmonic while the nonlinearity κ increases until they disappear at a certain critical value (in this case, $\kappa_{c}=4$). A further increase in κ leads to small oscillations around the left well, which is usually interpreted as a “self-trapping” effect that acts suppressing the tunneling effect. Therefore, this could explain Hund’s paradox.

### 7.2. Decoherence

As we commented at the beginning of this section, decoherence consists of the interaction between the system (a chiral molecule in this case) and the environment, making it possible to block the tunneling. In the pioneering works of Simonius [102] and Harris and Stodolsky [103], the authors used the density matrix formalism to introduce collisions between molecules which at the end tend to stabilize the molecule in one of the L or R states, concluding that “collisions or interactions with the medium tend to stabilize rather than destroy the asymmetry of the system” [103]. This conclusion has been reinforced over time; for example, in an important work of Trost and Hornberger [106], in which they showed how a collisional decoherence mechanism serves to stabilize states of chiral molecules. As they comment, “this stabilization can be understood in analogy to the quantum Zeno effect if one views the environment as continuously monitoring the molecular state.” [106].

Although there is a large variety of forms to study decoherence in chiral molecules, we will briefly comment on the coupling between a chiral molecule and a bath of harmonic oscillators [104,105]. Following Schlosshauer’s book [113], the total Hamiltonian consists of three parts: the system (the chiral molecule), the bath (harmonic oscillators), and the interaction between them. Therefore, the total Hamiltonian can be expressed as

(55) $$\hat{H}={\hat{H}}_{S}+{\hat{H}}_{B}+{\hat{H}}_{int},$$

where

(56) $$\begin{array}{cc} {\hat{H}}_{S} & =\delta {\hat{\sigma }}_{x}+\epsilon {\hat{\sigma }}_{z}, \end{array}$$

(57) $$\begin{array}{cc} {\hat{H}}_{B} & =\sum_{i}\left(\frac{1}{2m_{i}}{\hat{p}}_{i}^{2}+\frac{1}{2}m_{i}\omega_{i}^{2}{\hat{q}}_{i}^{2}\right), \end{array}$$

(58) $$\begin{array}{cc} {\hat{H}}_{int} & ={\hat{\sigma }}_{z}⊗\sum_{i}c_{i}{\hat{q}}_{i}. \end{array}$$

At this point, we have to comment on the three parts of the Hamiltonian. ${\hat{H}}_{S}$ is the two-level Hamiltonian (15), taking $E_{0}=0$ for simplicity. In ${\hat{H}}_{B}$, $m_{i}$ represents the mass of an harmonic oscillator, ${\hat{p}}_{i}$ its momentum, $\omega_{i}$ its frequency, and ${\hat{q}}_{i}$ its position, and the subindex *i* goes from 1 to *N*, *N* being the number of harmonic oscillators. Finally, we have to remark that, although we expose a specific interaction between the system and the bath (${\hat{H}}_{int}$), the interaction could be modeled using any combination of ${\hat{\sigma }}_{z}$, ${\hat{\sigma }}_{x}$ with ${\hat{p}}_{i}$, ${\hat{q}}_{i}$.

In order to show how the tunneling effect can be suppressed using decoherence theory, we consider a particular choice of variables, (z,ϕ), describing the system. That is, $c_{L}\left(t\right)$ and $c_{R}\left(t\right)$ can be written as $c_{L,R}\left(t\right)=\left|c_{L,R}\right|\left(t\right)e^{i\phi_{L,R}\left(t\right)}$. With this formalism in mind, the optical activity and the phase difference can be defined as $z\equiv |c_{R}\left(t\right){|}^{2}-{\left|c_{L}\left(t\right)\right|}^{2}$ and $\phi \left(t\right)\equiv \phi_{L}\left(t\right)-\phi_{R}\left(t\right)$ [110,111,112]. Using *z* and ϕ, we can express ${\hat{H}}_{S}$ in terms of two canonically conjugable variables, which act like the generalized momentum (*z*), and the generalized position (ϕ), relating classical and quantum mechanics. Using these canonically conjugable variables, a system-bath bilinear coupling can be introduced via a Caldeira–Legget-like approach [114,115,116], leading to the following total dimensionless Hamiltonian [110]:

(59) $$\hat{H}=-\sqrt{1-z^{2}}\cos\phi +\frac{\epsilon_{PV}}{\delta }z+\frac{1}{2}\sum_{i}\left(m_{i}{\hat{p}}_{i}^{2}+\frac{\omega_{i}^{2}}{m_{i}}{\hat{q}}_{i}^{2}\right)-\phi \sum_{i}c_{i}{\hat{q}}_{i}+\phi^{2}\sum_{i}c_{i}^{2}m_{i},$$

where the constants $m_{i}$, $c_{i}$, and $\omega_{i}$ are dimensionless couplings representing generalized masses, coupling with the environment and oscillator frequencies, respectively. We note that the last term of the Hamiltonian (59) acts as a renormalization term. After eliminating environmental degrees of freedom [117], and considering and Ohmic bath of oscillators (no memory), Hamilton’s equations of motion for z(t) and ϕ(t) can be expressed as

(60) $$\begin{array}{cc} \dot{z} & =-\sqrt{1-z^{2}}\sin\phi -\gamma \dot{\phi }+\xi \left(t\right), \end{array}$$

(61) $$\begin{array}{cc} \dot{\phi } & =\frac{z}{\sqrt{1-z^{2}}}\cos\phi +\frac{\epsilon_{PV}}{\delta }, \end{array}$$

where γ represents the damping constant and ξ(t) the noise term (an interested reader can see a more detailed exposition in [110]).

**Figure 8:**
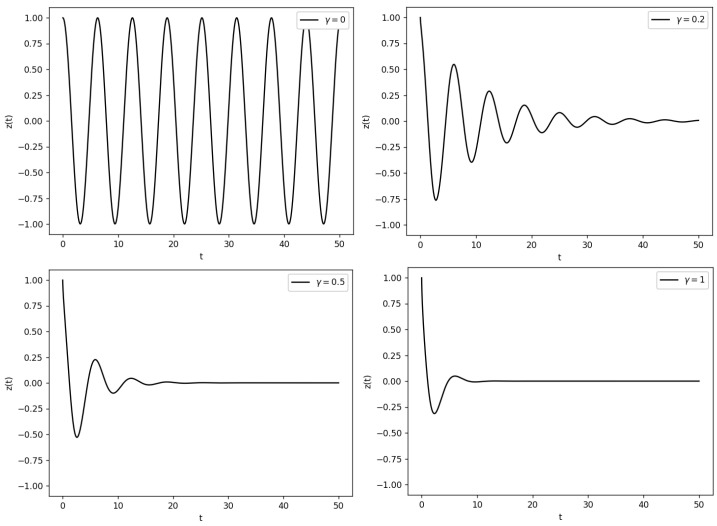
In this figure, the dissipative dynamics of the optical activity is represented for different damping constants, γ, observing the disappearance of tunneling. For simplicity, we have chosen ξ(t)=0 and $\epsilon_{PV}=0$.

According to Equations (60) and (61), the evolution of the optical activity follows the dynamics of a two-level system with damping and noise (similar to the Langevin equation), suppressing the tunneling oscillations [111], as can be seen in Figure 8.

Finally, we note that this formalism is also suitable for obtaining thermodynamic properties of the chiral system through a stochastic approach [111,112].

## 8. Discussion and Conclusions

Along this manuscript, we have reviewed the interplay between parity violation and quantum tunneling in the context of chiral molecules. Firstly, we have given a more theoretical point of view to finally end with a brief experimental overview.

By using a simple quantum treatment to describe chiral molecules as a two-level system, and after defining a good algebraic base to describe chiral molecules, the necessity of introducing quantum tunneling between the *L* and *R* enantiomers emerged. Interestingly, the tunneling time is inversely proportional to the energy difference between the time-independent Hamiltonian eigenfunctions with definite parity, |+〉 and |−〉, represented as δ in the manuscript. Subsequently, we have added parity violation as an essential ingredient of our model, which gives place to an energy difference between the *L* and *R* enantiomers, the so-called parity-violating energy difference (PVED). The introduction of this new parameter changes the probability of the chiral molecule to be in the *L* or *R* states, resulting in the apparent stability of one of the possible enantiomers of a chiral molecule when the PVED is substantially higher than δ. That is, when parity violation overcomes the tunneling effects, a possible solution to the “Hund’s paradox” emerges. Other possible solutions to Hund’s paradox are presented in the last section of this review. Once it is accepted that physical systems can interact with themselves or with the environment, it seems mandatory to include mean-field theory or decoherence theory in the model, both being able to suppress the tunneling effect. We think that it would be interesting to treat chiral molecules as an open system by combining high-level theoretical descriptions of both the molecule, the surroundings, and their interaction. Perhaps the medium can take a protagonistic role in order to enhance or diminish the PVED.

Throughout the rest of the manuscript, we mentioned possible parity-violating interactions, like axion interactions or modified gravity theories, focusing on electroweak interactions. Specifically, we commented on the electron–nucleon interaction mediated by weak neutral currents as it is the most used way to study the PVED in chiral molecules. Although we have a well established parity-violating Hamiltonian, calculating the PVED is difficult task. Different methods and approximations have been proposed based on different levels of the theory employed. We have remarked on some of them, such as the CIS-RHF formalism, the Breit–Pauli approximation, the Dirac–Fock theory, and the ZORA, DKH, and X2C methods. Finally, we have commented on some experimental searches of the PVED, noticing that important progress has been made over the last fifty years. Therefore, we think that we are finally close to detecting the PVED in chiral molecules.

## Data Availability

Data sharing not applicable. This article describes entirely theoretical research.
